# Supplementary material for: Pharmacokinetics/pharmacodynamics of gamithromycin for treating Pasteurella multocida infection in cattle using a tissue cage model
Source: PLoS One. 2025 May 29;20(5):e0323727. doi: 10.1371/journal.pone.0323727 (PMC12121915; doi:10.1371/journal.pone.0323727)
Supplement: S1 Table — (DOCX) [file pone.0323727.s001.docx]

**Pharmacokinetics/pharmacodynamics of gamithromycin for treating** Pasteurella multocida infection in cattle using a tissue cage model

Qingwen Yang^1^, Xuesong Liu^2^*, Yongzhi Lv^1^, Yushen Li^3^

**S1 Table: *In* *vitro* killing curve in serum.**

| **Time (h)** | **the density of the *Pasteurella multocida* (log_10_CFU/mL)** | | | | | | | | |
| --- | --- | --- | --- | --- | --- | --- | --- | --- | --- |
|  | **Control** | **0.25×MIC** | **0.5×MIC** | **1×MIC** | **2×MIC** | **4×MIC** | **8×MIC** | **16×MIC** | **32×MIC** |
| 0 | 6.00 | 6.00 | 6.00 | 6.00 | 6.00 | 6.00 | 6.00 | 6.00 | 6.00 |
| 2 | 7.81 | 7.80 | 7.60 | 7.21 | 5.96 | 5.96 | 5.41 | 5.25 | 5.31 |
| 4 | 7.97 | 7.92 | 7.80 | 7.26 | 5.82 | 5.82 | 5.06 | 4.89 | 4.80 |
| 6 | 8.06 | 7.97 | 8.02 | 7.27 | 5.21 | 5.21 | 3.38 | 3.28 | 3.05 |
| 8 | 8.20 | 8.14 | 7.89 | 6.61 | 4.68 | 4.68 | 2.23 | 2.19 | 2.16 |
| 10 | 8.27 | 8.21 | 8.10 | 5.71 | 3.90 | 3.90 | 2.21 | 2.17 | 2.13 |
| 12 | 8.28 | 8.27 | 8.30 | 5.11 | 2.81 | 2.81 | 2.17 | 2.16 | 2.11 |
| 24 | 8.05 | 7.95 | 7.84 | 4.45 | 2.47 | 2.47 | 2.15 | 2.14 | 2.12 |
