# Supplementary material for: Pharmacokinetics/pharmacodynamics of gamithromycin for treating Pasteurella multocida infection in cattle using a tissue cage model
Source: PLoS One. 2025 May 29;20(5):e0323727. doi: 10.1371/journal.pone.0323727 (PMC12121915; doi:10.1371/journal.pone.0323727)
Supplement: S2 Table — (DOCX) [file pone.0323727.s002.docx]

**Pharmacokinetics/pharmacodynamics of gamithromycin for treating** Pasteurella multocida infection in cattle using a tissue cage model

Qingwen Yang^1^, Xuesong Liu^2^*, Yongzhi Lv^1^, Yushen Li^3^

**S2 Table: *In* *vitro* killing curve in MHB.**

| **Time (h)** | **the density of the *Pasteurella multocida* (log_10_CFU/mL)** | | | | | | | | |
| --- | --- | --- | --- | --- | --- | --- | --- | --- | --- |
|  | **Control** | **0.25×MIC** | **0.5×MIC** | **1×MIC** | **2×MIC** | **4×MIC** | **8×MIC** | **16×MIC** | **32×MIC** |
| 0 | 6.00 | 6.00 | 6.00 | 6.00 | 6.00 | 6.00 | 6.00 | 6.00 | 6.00 |
| 2 | 7.89 | 7.85 | 7.72 | 7.72 | 6.20 | 5.55 | 5.53 | 5.41 | 5.50 |
| 4 | 8.00 | 7.93 | 7.81 | 7.78 | 5.93 | 5.41 | 5.28 | 5.07 | 4.87 |
| 6 | 8.36 | 8.33 | 8.25 | 8.21 | 5.32 | 4.71 | 2.31 | 2.28 | 2.27 |
| 8 | 8.28 | 8.23 | 8.12 | 8.10 | 4.80 | 3.69 | 2.27 | 2.25 | 2.23 |
| 10 | 8.41 | 8.30 | 8.18 | 8.15 | 4.16 | 2.32 | 2.24 | 2.19 | 2.18 |
| 12 | 8.51 | 8.43 | 8.25 | 8.21 | 2.62 | 2.28 | 2.21 | 2.17 | 2.16 |
| 24 | 8.26 | 8.22 | 8.12 | 8.09 | 2.38 | 2.23 | 2.20 | 2.16 | 2.14 |
