# Supplementary material for: Pharmacokinetics/pharmacodynamics of gamithromycin for treating Pasteurella multocida infection in cattle using a tissue cage model
Source: PLoS One. 2025 May 29;20(5):e0323727. doi: 10.1371/journal.pone.0323727 (PMC12121915; doi:10.1371/journal.pone.0323727)
Supplement: S3 Table — (DOCX) [file pone.0323727.s003.docx]

**Pharmacokinetics/pharmacodynamics of gamithromycin for treating** Pasteurella multocida infection in cattle using a tissue cage model

Qingwen Yang^1^, Xuesong Liu^2^*, Yongzhi Lv^1^, Yushen Li^3^

**S3 Table: The gamithromycin concentration in serum after intravenous injection.**

| **Time (h)** | **Concentration (ng/mL)** |
| --- | --- |
| 0.083 | 5209.66 |
| 0.17 | 2515.32 |
| 0.25 | 1855.99 |
| 0.5 | 923.87 |
| 0.75 | 505.74 |
| 1 | 287.5 |
| 2 | 183.61 |
| 3 | 117.02 |
| 6 | 88.85 |
| 9 | 75.18 |
| 12 | 66.25 |
| 24 | 46.12 |
| 48 | 21.59 |
| 72 | 13.22 |
| 96 | 5.95 |
| 120 | 5.49 |
| 144 | 4.49 |
| 168 | 3.8 |
| 192 | 2.23 |
