# Supplementary material for: Pharmacokinetics/pharmacodynamics of gamithromycin for treating Pasteurella multocida infection in cattle using a tissue cage model
Source: PLoS One. 2025 May 29;20(5):e0323727. doi: 10.1371/journal.pone.0323727 (PMC12121915; doi:10.1371/journal.pone.0323727)
Supplement: S4 Table — (DOCX) [file pone.0323727.s004.docx]

**Pharmacokinetics/pharmacodynamics of gamithromycin for treating** Pasteurella multocida infection in cattle using a tissue cage model

Qingwen Yang^1^, Xuesong Liu^2^*, Yongzhi Lv^1^, Yushen Li^3^

**S4 Table: The gamithromycin concentration in transudate and exudate samples after intravenous injection.**

| **Time (h)** | **Concentration (ng/mL)** | |
| --- | --- | --- |
|  | **Transudate** | **Exudate** |
| 1 | 41.48 | 62.78 |
| 3 | 91.5 | 87.14 |
| 6 | 67.35 | 109.02 |
| 9 | 47.68 | 63.82 |
| 12 | 39.5 | 55.18 |
| 24 | 19.01 | 40.41 |
| 48 | 14.91 | 18.48 |
| 72 | 11.96 | 20.27 |
| 96 | 10.09 | 13.47 |
| 120 | 7.5 | 9.91 |
| 144 | 6.61 | 8.21 |
| 168 | 4.88 | 6.65 |
| 192 | 3.09 | 3.77 |
