# Supplementary material for: Pharmacokinetics/pharmacodynamics of gamithromycin for treating Pasteurella multocida infection in cattle using a tissue cage model
Source: PLoS One. 2025 May 29;20(5):e0323727. doi: 10.1371/journal.pone.0323727 (PMC12121915; doi:10.1371/journal.pone.0323727)
Supplement: S5 Table — (DOCX) [file pone.0323727.s005.docx]

**Pharmacokinetics/pharmacodynamics of gamithromycin for treating** Pasteurella multocida infection in cattle using a tissue cage model

Qingwen Yang^1^, Xuesong Liu^2^*, Yongzhi Lv^1^, Yushen Li^3^

**S5 Table: The gamithromycin concentration in serum after subcutaneous injection.**

| **Time (h)** | **Concentration (ng/mL)** |
| --- | --- |
| 0.083 | 178.69 |
| 0.17 | 254.05 |
| 0.25 | 331.1 |
| 0.5 | 333.47 |
| 0.75 | 372.03 |
| 1 | 384.26 |
| 2 | 363.51 |
| 3 | 338.51 |
| 6 | 208.99 |
| 9 | 147.57 |
| 12 | 95.11 |
| 24 | 55.43 |
| 48 | 31.27 |
| 72 | 14.16 |
| 96 | 11.17 |
| 120 | 8.19 |
| 144 | 6.00 |
| 168 | 4.33 |
| 192 | 3.49 |
