# Supplementary material for: Pharmacokinetics/pharmacodynamics of gamithromycin for treating Pasteurella multocida infection in cattle using a tissue cage model
Source: PLoS One. 2025 May 29;20(5):e0323727. doi: 10.1371/journal.pone.0323727 (PMC12121915; doi:10.1371/journal.pone.0323727)
Supplement: S6 Table — (DOCX) [file pone.0323727.s006.docx]

**Pharmacokinetics/pharmacodynamics of gamithromycin for treating** Pasteurella multocida infection in cattle using a tissue cage model

Qingwen Yang^1^, Xuesong Liu^2^*, Yongzhi Lv^1^, Yushen Li^3^

**S6 Table: The gamithromycin concentration in transudate and exudate samples after subcutaneous injection.**

| **Time (h)** | **Concentration (ng/mL)** | |
| --- | --- | --- |
|  | **Transudate** | **Exudate** |
| 1 | 23.98 | 54.99 |
| 3 | 34.54 | 77.16 |
| 6 | 42.42 | 84.56 |
| 9 | 44.01 | 88.68 |
| 12 | 34.45 | 41.53 |
| 24 | 13.34 | 26.35 |
| 48 | 12.64 | 19.65 |
| 72 | 8.25 | 11.97 |
| 96 | 5.31 | 9.53 |
| 120 | 5.47 | 7.63 |
| 144 | 3.75 | 4.7 |
| 168 | 3.12 | 3.84 |
| 192 | 3.3 | 3.13 |
