# Supplementary material for: Pharmacokinetics/pharmacodynamics of gamithromycin for treating Pasteurella multocida infection in cattle using a tissue cage model
Source: PLoS One. 2025 May 29;20(5):e0323727. doi: 10.1371/journal.pone.0323727 (PMC12121915; doi:10.1371/journal.pone.0323727)
Supplement: Attachment — Submitted filename: Response to Reviewers.docx [file pone.0323727.s007.docx]

Dear Reviewers,

Thank you for your letter and for the reviewers' comments concerning our manuscript. Those comments are all valuable and helpful for revising and improving our paper, as well as the important guiding significance to our researches. We have studied comments carefully and have made correction which we hope meet with approval. Revised portion are marked in the paper. The main corrections in the paper and the responds to the reviewer's comments are as flowing:

Reviewer #1: To summarize, the study investigates the PK/PD parameters of gamithromycin for the treatment of BRD by *Pasteurella multocida* by examining the serum, transudates and exudates of 2 groups of cattle (3 each in a group, two way cross over, by 6mg/kg i.v and .c injection), using tissue cage method. The study shows that overall s.c. injection has higher Cmax, Tmax, T1/2b, Vd and slower clearance compared to i.v. injection.
Minor issue: The authors have not specified how the parameters are expressed in tables 2 and 3 (mean+/-SEM) and n=6 or 3?

Response:

The PK parameters were conducted using WinNonlin software and presented as mean±SD. The n=6.

Reviewer #2: The pharmacokinetics and pharmacodynamics of the Azalide drug, Gamithromycin has been determined.
1- what is the control/ reference drug used along with gamithromycin to compare all the measured properties.
Response:

Thank you for your comment regarding the control/reference drug in our study . We acknowledge that we did not specifically set a traditional control drug in the experiment. However, we compared the measured properties of gamithromycin with those from numerous previously published articles on this drug.

These references provided valuable data on various aspects such as its pharmacokinetic parameters and pharmacodynamic characteristics in different experimental settings and populations. By conducting this comprehensive literature review and comparison, we aimed to place our findings within the context of existing knowledge about gamithromycin and ensure the reliability and relevance of our results.

2- The gamithromycin drug purchased from a particular company, how is that specific company selected and at which basis.

Response: Gamithromycin was developed by Merial in France, and they developed it into an injectable solution. Therefore, the injectable gamithromycin was purchased from Merial in France. Tiaozhan Biotechnology obtained the New Veterinary Drug Registration Certificate of the People's Republic of China for gamithromycin in 2019 and has won the National Science and Technology Progress Award. Therefore, the gamithromycin standard was purchased from this company.

3- The drug concentrations or levels are measured at different time intervals. Is any consideration is given to the fact that inoculum of the microorganism is also standardized at various time intervals. How is it made possible to keep the concentration of the inoculum uniform at various time intervals.

Response:

We determined the concentration of bacteria and plotted the bacterial growth curve through preliminary experiments, and ensured that the inoculum amount in each experiment was standardized by means of these preliminary experiments.

4- the ATCC or other specific number/lot of the *Pasteurella multocida* is not mentioned. How is it confirmed that the particular strain of *Pasteurella multocida* is susceptible, resistant or indeterminate?

Response:

Due to our negligence, it was not clearly stated in the article that we used *Streptococcus pneumoniae* ATCC 49619 as the quality control strain for the MIC test. We have corrected this error in the article.

Yours sincerely,

Xuesong Liu
