# Supplementary material for: Pharmacokinetics/pharmacodynamics of gamithromycin for treating Pasteurella multocida infection in cattle using a tissue cage model
Source: PLoS One. 2025 May 29;20(5):e0323727. doi: 10.1371/journal.pone.0323727 (PMC12121915; doi:10.1371/journal.pone.0323727)
Supplement: Attachment — Submitted filename: Pharmacokinetics.docx [file pone.0323727.s008.docx]

Pharmacokinetics/pharmacodynamics of gamithromycin for treating Pasteurella multocida infection in cattle using a tissue cage model

The study investigates the PK/PD of gamithromycin against Pasteurella multocida in cattle using a tissue cage model. The aim is to optimize dosing regimens for bovine respiratory disease (BRD). The research question is relevant, and the use of a tissue cage model is appropriate. However, there are several areas where the manuscript needs improvement in clarity, detail, and justification of methodological choices.

Points need explanation

Inconsistent Units and Presentation of PK/PD Data: The authors repeatedly talk about "AUC0-24h/MIC values divided by 24 h." This is confusing and unconventional. While the authors claim it makes the value dimensionless and easier to interpret, it's essentially just presenting an average concentration over 24 hours relative to the MIC. Instead of this non-standard practice, the authors need to present the actual AUC0-24h/MIC values and provide clear rationale for their target selection (e.g., based on similar studies with macrolides). The approach of dividing by 24 h introduces unnecessary confusion and departs from standard PK/PD reporting. This needs to be revised to follow conventional practices.

Justification for 6 mg/kg Dose: The choice of 6 mg/kg as the gamithromycin dose is never justified. Is this the standard clinical dose? If so, it should be stated. If not, the rationale for choosing this dose needs to be explained, considering its relevance to clinical application and existing literature.

Lack of Detail in Statistical Analysis: The description of statistical analysis is vague. The authors mention t-tests and Bonferroni's correction but do not specify what was compared using the t-tests. Were the IV and SC routes directly compared in terms of PK parameters? Were the bacterial counts compared at different time points? More clarity is needed.

Minor Concerns:

Introduction - Clarity & Flow: The introduction could be more focused. The sentences "When using AUC/MIC value for evaluating breakpoints, a time dimension (in hours) is included, often presenting difficulties for reader comprehension and discussion among scientists. However, division of the AUC/MIC value by the time duration produces more easily understandable values." is not clear.

Materials and Methods - Strain Information: More information about the Pasteurella multocida NM-5-7 strain should be provided. Was it a field isolate? What was the source?

Results - Clarity of Ex Vivo Results: The description of the ex vivo time-kill curves is a bit convoluted. It's difficult to follow which time points showed inhibitory or bactericidal activity. A table summarizing these results might improve clarity.

Figures - Labeling: Check all figures for clear labeling of axes and legends.

Discussion - Acknowledge Limitations: The discussion section acknowledges some limitations, but it could be expanded. For example, the tissue cage model is an artificial system, and the results may not perfectly translate to a real-world BRD infection in the lungs.

In this study, the bioavailability following s.c. administration was found to be 112.95%, which was similar to the value found in pigs (117.6%) and higher than those reported in broiler chickens (102.4%) and rabbits (86.7%). These discrepancies may be due to species differences.

These finding need references .

Recommendations:

Address the Bioavailability Issue: Thoroughly re-evaluate the data and calculations related to bioavailability. Provide a clear explanation for any observed values exceeding 100% or correct the error.

Revise PK/PD Presentation: Present the AUC0-24h/MIC values directly, without dividing by 24 h. Justify the choice of target AUC/MIC values based on existing literature and PK/PD principles.

Justify Dose Selection: Provide a clear rationale for choosing the 6 mg/kg dose of gamithromycin.

Clarify Statistical Analysis: Provide more detail about the statistical methods used and what comparisons were made.

Improve Clarity and Flow: Revise the introduction and results sections for improved clarity and readability.

Provide More Detail: Add more information about the bacterial strain, animal welfare, and limitations of the study.

Minor mistake that should be checked through out the article

Here are some key mistakes and areas for improvement in your document:

**General Issues:**

**Grammar and Typographical Errors:**

There are several minor grammatical errors, typos, and inconsistent phrasing throughout the document (e.g., "randomlyy" instead of "randomly" and "received received" in "each animal receiving received gamithromycin").

**Inconsistent Formatting:**

Some abbreviations are introduced without explanation, while others are fully spelled out multiple times (e.g., "PK/PD" vs. "pharmacokinetics/pharmacodynamics").

In some sections, drug and bacterial names are italicized (e.g., *Pasteurella multocida*), but in other areas, they are not.

**Redundant or Repetitive Sentences:**

Some points are unnecessarily repeated, such as explaining PK/PD modeling more than once in different sections.

The phrase **"the LOD was 200 CFU/mL"** appears multiple times unnecessarily.

**Sentence Structures:**

Example: *"However, to the best of our knowledge, there is no information on the combination of in vitro PK data with ex vivo PD values for predicting the various antibacterial activities of gamithromycin in a cattle tissue cage model."*
→ This sentence is too long and would be clearer if broken down.

**Section-Specific Issues:**

**Abstract:**

The values for **AUC0-24h/MIC** should be consistently formatted (e.g., missing the word "were" in *"while for bactericidal activity, the AUC0-24h/MIC values over 24 h in sera and exudates 3.76 and 5.31, respectively"* → should be **"...values were 3.76 and 5.31, respectively"**).

The abstract should provide more context on **why** gamithromycin is significant.

**Introduction:**

**Sentence clarity:**

"Gamithromycin was developed for preventing and treating BRD resulting from *P. multocida*, *Mannheimia haemolytica*, and *Histophilus somni* infections."
→ Better phrased as: *"Gamithromycin was developed to prevent and treat BRD caused by P. multocida, M. haemolytica, and H. somni."*

**Unnecessary repetition:**

PK/PD models are explained twice, once in general and again in the same section.

**Materials and Methods:**

**Inconsistencies in units and spacing:**

"Cattle (mean weight ± SD, 142±18 kg)" → There should be spaces around the ± sign (should be: **"142 ± 18 kg"**).

**Missing references:**

Some methodologies are referenced (e.g., [16, 17]), but others lack proper citations.

**Results:**

**Inconsistent Number Formatting:**

In PK values, spaces between units should be standardized (e.g., "AUC0-last was 5.52 μg·h/mL" should match formatting throughout).

**Table and Figure References:**

"Figure 1" and "Table 1" should be consistently formatted (sometimes written as **Fig. 1** and **Table 1**).

Conclusion:

This study has the potential to provide valuable insights into the PK/PD of gamithromycin for treating Pasteurella multocida infections in cattle. However, addressing the major concerns outlined above is crucial to improve the rigor, clarity, and overall impact of the research.
