# Supplementary material for: Pharmacokinetics/pharmacodynamics of gamithromycin for treating Pasteurella multocida infection in cattle using a tissue cage model
Source: PLoS One. 2025 May 29;20(5):e0323727. doi: 10.1371/journal.pone.0323727 (PMC12121915; doi:10.1371/journal.pone.0323727)
Supplement: Attachment — Submitted filename: Response_to_Reviewers_auresp_2.docx [file pone.0323727.s009.docx]

Dear Reviewer,

Thank you for your letter and for the reviewers' comments concerning our manuscript. Those comments are all valuable and helpful for revising and improving our paper, as well as the important guiding significance to our researches. We have studied comments carefully and have made correction which we hope meet with approval. Revised portion are marked in the paper. The main corrections in the paper and the responds to the reviewer's comments are as flowing:

Reviewer #1: I congratulate the authors for presenting the study in a detailed manner. The authors have clearly explained the methodology and results of the study and addressed the issues raised. The limitations of the study and the direction for future research have also been explained clearly.
Minor issue: The authors conclude that the lower target PD values will prompt dosage reductions during calculations. (Line 276) It would be helpful, (if possible) if the authors could propose based on the current study findings, a dosing regimen compared to the current regimen, which could be used for a future comparison trial to determine the correct dosing regimen.

Response: Based on the current study, the dose of gamithromycin required to achieve a bactericidal effect via subcutaneous administration is 6.04 mg/kg, which is close to the officially recommended dose. However, this dose calculation is based on the MIC value of a single strain rather than the MIC_90_ value. The MIC_90_ represents the minimum drug concentration required to inhibit the growth of 90% of the tested strains and is generally considered a more reliable and clinically representative pharmacodynamic parameter. Since the susceptibility of different strains to the drug may vary significantly, relying solely on the MIC value of a single strain may lead to limitations in dose recommendations.

In the future, we will conduct more in-depth research and use MIC_90_ as an indicator for dose calculation.

Yours sincerely,

Xuesong Liu
